# Malignant transformation of a non-healing traumatic wound on the lower extremity: A case report

**DOI:** 10.1016/j.ijscr.2018.11.026

**Published:** 2018-11-22

**Authors:** Deborah-Leigh Day, Wahida Chakari, Steen Henrik Matzen

**Affiliations:** a Department of Plastic Surgery and Breast Surgery, Zealand University Hospital, Sygehusvej 10, 4000, Roskilde, Denmark; b Department of Plastic, Reconstructive and Burns Surgery, Copenhagen University Hospital, Blegdamsvej 9, 2100, Copenhagen, Denmark

**Keywords:** Basal cell carcinoma, Marjolin’s ulcer, Chronic wounds, Malignant ulcer

## Abstract

Objective: Malignant transformation of chronic non-healing wounds is often overlooked. If a chronic wound fails to heal, or if there are changes in the characteristics of a wound, a biopsy should be performed.

Methods: This case is presented in line with SCARE guidelines.

Presentation of case: We present a case of a man with a non-healing traumatic wound over two years in which a basal cell carcinoma was diagnosed by biopsy. The basal cell carcinoma was radically excised and the defect was closed using a split thickness skin transplant.

Discussion: The term Marjolin’s ulcer, pathophysiology and recommended treatment.

Conclusion: Delayed diagnosis of malignancy in chronic wounds can lead to a worsened prognosis and therefore it is important to exclude a malignant aetiology. Malignant transformation of chronic wounds is not common but Marjolin's ulcer should be considered.

## Highlights

• It is important to exclude malignancy in chronic wounds.
• Change in wound characteristic should be investigated with a biopsy.
• Marjolin’s ulcer refers most commonly to squamous cell carcinoma arising in scar tissue.
• Basal cell carcinoma, sarcoma and melanoma are also described in Marjolin’s ulcers.

## 1 Introduction

Basal cell carcinoma (BCC) is the most common non-melanoma (NMM) skin cancer [1]. BCCs seldom metastasize, but can be locally invasive [2]. BCCs are suspected during clinical examination with the use of dermatoscopy of the lesion and diagnosis is confirmed histologically. The pathogenesis of BCC is multifactorial. Risk factors include exposure to ultraviolet light, ionizing radiation, chemicals such as arsenic, immunosuppression and phenotypical factors, for example light skin, light and red hair colour and light eye colour. Genetic factors also play a role [1].

Trauma has also been suggested as a risk factor in the development of basal cell carcinomas [3,4]. Malignant degeneration of wounds and scars is a well-accepted concept, but the pathophysiology has been much debated. The earliest reports were noted by Celcus in the 1th century in burn scars [5]. Jean-Nicholas Marjolin described ulceration within a burn wound in 1828. In 1850 Robert Smith described the malignant nature of the pathology Jean-Nicholas Marjolin had reported and called it Marjolin’s ulcer (MU). The term MU is most often used when referring to burn scar wounds and squamous cell carcinomas (SCC) [6]. Other types of carcinomas, for example BCC, sarcoma and melanoma, arising in scars/chronic wounds have been called scar tissue carcinomas [7]. The term MU is however often used to describe malignancy that arises in chronic wounds and cutaneous scars.

We present a case of a 73-year-old man with a history of a non-healing wound in which a BCC is diagnosed. The patient was treated at the Department of Plastic Surgery and Breast Surgery, Zealand University Hospital, Roskilde, Denmark. This case highlights the importance of considering and excluding malignancy in a chronic wound. The work has been reported in line with SCARE guidelines [8].

## 2 Case report

A 71-year-old man, known with polycythemia vera and aortastenosis, sustained a wound laterally on his right mid lower leg when he fell and struck a hospital bed, which failed to heal over a 2-year period. The patient was referred 2 years post trauma to a wound centre as the wound had failed to heal after 6 months of treatment by his General Practitioner.

**Fig. 1:**
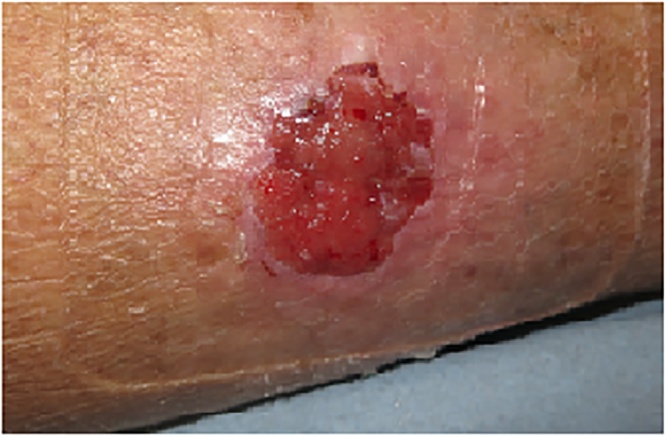
Non-healing wound on the right lower limb approximately 4 cm in diameter.

At the wound centre the patient received treatment for a non-healing ulcer by means of various ointments, honey and dressings, with a view to referral for skin transplantation if the wound did not heal satisfactorily. It is noted that the patient was not diabetic and did not have any cardiovascular risk factors but was undergoing follow-up for aortastenosis and polycythemia vera. There was initially improvement in the wound over an approximately six-month period, but due to the return of hypergranulation tissue, increasing size of the wound and a request from the patient, a punch biopsy of the wound was taken which confirmed basal cell carcinoma. The patient was referred to The Department of Plastic Surgery and Breast Surgery, Roskilde Hospital for excision of the carcinoma. The ulcer, approximately 4 cm in diameter (Fig. 1), was surgically excised with a 5 mm margin, without the use of frozen sections, and covered with a split thickness skin transplant. The histology showed an ulcerated basal cell carcinoma of nodular type, with growth deep into the dermis, but not involving the subcutaneous tissue. Histology also showed that the BCC was radically removed. Post-operative control included follow up in the Plastic Surgery clinic for approximately one month. After the skin transplant healed the patient was discharged from follow up in our clinic to yearly control at his Dermatologist. There has been no local recurrence of the BCC. The patient was subsequently diagnosed, with diabetes mellitus type two (DMT2) and started treatment with dietary changes and exercise.

## 3 Discussion

MU are reported as arising within about 2% of all burn scars but are also described within surgical scars and chronic wounds with varying aetiologies. Traumatic wounds, diabetic wounds and chronic venous ulcer wounds have also been reported [9].

MU most commonly refer to SCC, however, BCC, melanoma and sarcoma have also been described. In a literature review by Kowal et al [10] 71% of MUs were SCC, 12% BCC and 6% melanoma.

In the literature MUs are described as either chronic or acute. Most cases reported are chronic with an average latency period from time of injury to malignant transformation of about 32 years [10]. Acute MUs arise within 12 months. There is however a debate about acute MU. Some authors argue that the presence of skin cancer within an acute wound could be coincidental. Other authors state that the injury accelerates an already present process of carcinogenesis. It is not clear from the literature if there is histological or prognostic difference between acute and chronic MU [11]. What is clear is that changes in wound character or chronicity of a wound should lead to a biopsy. In an article by Bazalinksi et al. [12] it is reported that SCC are more common in chronic MU and BCC in acute MU.

The patient in our case was diagnosed with BCC around two years after trauma. Furthermore, the patient was diagnosed with DMT2 after surgery. It is known that patients with DMT2 have delayed wound healing. These patients are at risk for developing chronic ulcers, which is thought to be a risk factor for malignant transformation [11]. DMT2 has been showed to be a risk factor for certain types of cancer and has been linked in the literature with non-melanoma skin cancer [13]. There is however no evidence for a causal link. It must also be noted that the patient presented in this case is known with Polycythemia Vera. In 2012 he began treatment with Ruxolitinib, a Janus-associated kinase inhibitor. In a study by Blecham et al. [14] patients who have received Ruxolitinib are reported to develop aggressive skin cancers, including BCC and SCC. Willkens et al. [15] also describe the development of BCC in a patient treated with Ruxolitinib but do not ascribe a causal link. Further research needds to be done to determine a causal link. In our case there are multiple factors that could potentially have caused the onset of carcinogenesis. In the literature the pathogenesis of MU is unknown, however multiple theories have been postulated [11]. These include:

1 toxin theory in which tissue damage leads to the release of toxins which can cause cell mutation
2 chronic irritation theory where by chronic irritation induces carcinogenesis
3 injury stimulates latent malignant cells within the wound
4 the avascular nature of scar tissue allows unchecked tumor growth
5 a genetic theory involving the p53 gene, HLA DLA and FAS genes.

The recommended treatment of MU is wide local excision and lymph node dissection. Most cases describe recommendations for MU of SCC type. The term MU covers a variety of cancers and there is need for further research so that treatment recommendations can be given for the various tumors found in scars and chronic non-healing wounds.

## 4 Conclusion

We have presented a patient in whom a non-healing traumatic wound masks a BCC. It is essential to be aware of the multifactorial nature of chronic wounds and this case highlights the importance of excluding malignancy in all non-healing wounds. Wounds that are chronic or change in characteristic should be biopsied to exclude malignancy.

## Conflicts of interest

None.

## Funding

None.

## Ethical approval

The Zealand University Hospital exempts case reports from ethical approval.

## Consent

Written informed consent was obtained from the patient for the publication of this case report and accompanying images. A copy of the written consent is available for review from the Editor-in -Chief of this journal on request.

## Author contribution

Deborah-Leigh Day: The conception and design of the study, collection of data and writing: original draft.

Wahida Chakari: The conception and design of the study and writing: review and editing.

Steen Henrik Matzen: Critical revision of the article.

## Registration of research studies

Not applicable.

## Guarantor

Deborah-Leigh Day.

## Provenance and peer review

Not commissioned externally peer reviewed.
